# Dental and Skeletal Side Effects of Oral Appliances Used for the Treatment of Obstructive Sleep Apnea and Snoring in Adult Patients—A Systematic Review and Meta-Analysis

**DOI:** 10.3390/jpm12030483

**Published:** 2022-03-16

**Authors:** Ioannis A. Tsolakis, Juan Martin Palomo, Stefanos Matthaios, Apostolos I. Tsolakis

**Affiliations:** 1 Department of Orthodontics, School of Dentistry, Aristotle University of Thessaloniki, 541 24 Thessaloniki, Greece; 2 Department of Orthodontics, Case Western Reserve University School of Dental Medicine, Cleveland, OH 44106, USA; palomo@case.edu (J.M.P.); stefmattheos@hotmail.com (S.M.); 3 Department of Orthodontics, School of Dentistry, National and Kapodistrian, University of Athens, 157 72 Athens, Greece; apostso@otenet.gr

**Keywords:** mandibular advancement devices (MADs), obstructive sleep apnea (OSA), dental effects, skeletal effects, adults

## Abstract

Background: Mandibular advancement devices for obstructive sleep apnea treatment are becoming increasingly popular among patients who do not prefer CPAP devices or surgery. Our study aims to evaluate the literature regarding potential dental and skeletal side effects caused by mandibular advancement appliances used for adult OSA treatment. Methods: Electronic databases were searched for published and unpublished literature along with the reference lists of the eligible studies. Randomized clinical trials and non-randomized trials assessing dental and skeletal changes by comparing cephalometric radiographs were selected. Study selection, data extraction, and risk of bias assessment were performed individually and in duplicate. Fourteen articles were finally selected (two randomized clinical trials and 12 non-randomized trials). Results: The results suggest that mandibular advancement devices used for OSA treatment increase the lower incisor proclination by 1.54 ± 0.16°, decrease overjet by 0.89 ± 0.04 mm and overbite by 0.68 ± 0.04 mm, rotate the mandible downward and forward, and increase the SNA angle by to 0.06 ± 0.03°. The meta-analysis revealed high statistical heterogeneity. Conclusions: The MADs affect the lower incisor proclination, overjet, overbite, the rotation of the mandible and the SNA angle. More randomized clinical trials providing high-quality evidence are needed to support those findings.

## 1. Introduction

Obstructive sleep apnea syndrome (OSAS) is a sleep breathing disorder characterized by a periodic collapse of the upper airway during sleep. OSAS is diagnosed when there are five or more obstructive respiratory events per hour of sleep and signs/symptoms (i.e., snoring, and daytime sleepiness) or related medical/psychiatric disorders (i.e., hypertension). A sleep breathing disorder can also be considered as obstructive sleep apnea when 15 or more respiratory events occur in an hour of sleep without any signs/symptoms or disorders [1]. Although snoring is its primary symptom, some patients have less than five respiratory events per hour of sleep, and thus they are considered non-apnoeic snorers [2]. Respiratory events include obstructive and mixed apneas, hypopneas, and respiratory effort-related arousals, according to the American Academy of Sleep Medicine (AASM).

OSAS prevalence is high in adults, as it is thought to affect 14% of men and 5% of women. Its consequences, such as cardiovascular conditions, neurocognitive and mental health problems, decrease patients’ quality of life and can be lethal in some cases [3,4,5,6].

OSAS therapies include conservative measures (i.e., weight loss, better sleeping position, and alcohol avoidance), upper airway surgery, nasal continuous positive airway pressure (CPAP), and oral appliances [6]. Although CPAP, a device that continuously pressures the upper airway and prevents its collapse during sleep, is considered the gold standard for obstructive sleep apnea (OSA) treatment, oral appliances can be used as an alternative, and are often preferred by the patients. Oral appliances (OAs) are also proposed for apnoeic patients with intolerance to CPAP or non-apnoeic snorers who have failed conservative lifestyle changes (i.e., weight loss) [7].

Nearly 100 different oral appliances are currently available, and they can be divided into three main groups: mandibular advancement devices (MADs), tongue retaining devices (TRD), and soft palate lifting devices. All of them intend to maintain the airway open, preventing its collapse. MADs which are the most commonly used, advance the mandible in order to increase the airway space and reduce pharyngeal collapsibility [8].

Custom, titratable MADs are the most effective OAs for OSAS and snoring, according to the AASM. These MADs can reduce the apnea-hypopnea index (AHI), oxygen desaturation, arousal index, and increase oxygen saturation, although to a lower extent than CPAP in patients with OSAS. On the other hand, they have equivalent effectiveness compared with CPAP in the reduction in daytime sleepiness and hypertension and quality of life improvement. Furthermore, they have greater device adherence and less possibility of treatment discontinuation due to side effects (odds ratio of discontinuation of treatment due to the use of an OA vs. CPAP: 0.54:1). These oral appliances can also be useful in primary snoring, as they improve sleep quality and quality of life (QOL) and reduce snoring frequency and intensity [9,10].

In the past decades, many studies have examined the adverse effects of oral appliance use in OSAS/snoring treatment. These include subjective side effects, such as mouth dryness and temporomandibular dysfunction, examined through questionnaires and objective side effects assessed by dental casts and cephalometric analysis [11,12].

To our knowledge, there are some literature reviews, assessing the dental and skeletal side effects of mandibular protruding devices for the treatment of adult obstructive sleep apnea and snoring. In 2004, Hoekema et al. stated that there were predominantly occlusal changes, but they could not conclude about long term side effects [13]. More recently, Araie et al. (2018) found significant dental changes, regarding overjet and overbite decrease and lower incisor axis-mandibular plane angle (L1-MP) increase, but no skeletal changes [14]. Patel et al. (2019) also reported that there was a significant reduction in overjet and overbite [15]. On the other hand, Bartolucci et al. (2019) were the first to report significant skeletal changes in point A-nasion-point Β angle (ANB) and anterior facial height, except for dental changes in overjet, overbite, and incisor inclination [16]. Moreover, Mendes Martins et al. (2019) concluded that there were mainly long-term dental changes. The treatment duration and the population sample in some of the included studies for these reviews were small [17]. Furthermore, a cephalometric analysis, for assessing skeletal changes was not performed in all their included studies. A cephalometric analysis is based on the lateral X-ray tracing anatomic landmarks. The angles of these anatomic landmarks can conclude in valuable and accurate information for the skeletal and dental changes. Some of the most important landmarks for this research are the SNA angle that refers to the relationship of maxilla to the cranial base, the SNB angle that reveals the relationship of mandible to the cranial base, the ANB that refers to the relationship between maxilla and mandible, and the L1-MP angle that refers to the angulation of the lower incisors to the mandibular plane.

Our study aims to systematically review the most up-to-date scientific literature related with dental and skeletal changes caused by mandibular advancement devices used for the adult OSAS/snoring treatment, and perform a meta-analysis, in order to strengthen the current knowledge and help sleep physicians and qualified dentists/orthodontists to improve treatment’s efficacy and prevent discontinuation due to side effects.

## 2. Materials and Methods

### 2.1. Protocol and Registration

The protocol for this present systematic review was registered on the National Institute of Health Research Database (Protocol: CRD42020169736).

### 2.2. Eligibility Criteria

The following selection criteria were applied for the review:

1. Study design: randomized clinical trials (RCTs), quasi-randomized clinical trials, and non-randomized prospective and retrospective trials (non-RCTs), without any restriction in language and time of publication, were considered eligible for inclusion in this review;
2. Participants: adult patients with obstructive sleep apnea syndrome or snoring;
3. Interventions: studies that treated obstructive sleep apnea and/or snoring patients with an oral appliance that protruded the mandible forward;
4. Comparisons: comparisons were made between baseline and follow-up patient characteristics;
5. Outcomes measures: any objective dental and skeletal change, in the treated patients.

### 2.3. Information Sources, Search Strategy and Study Selection

A literature search was carried out in the following electronic databases: Medline database (via PubMed), Embase (via Ovid), Scopus, CENTRAL, Google Scholar, and the Cochrane Oral Health Group’s Trial Register. Language restrictions were not applied. Unpublished literature was searched on ClinicalTrials.gov (accessed on 24 February 2022) and the National Research Register. The Medical Subject Heading (MeSH) terms used for this study were “sleep apnea syndromes”, “adverse effects”, “jaw”, and “tooth”. Conference proceedings and abstracts were also accessed when possible. The authors were contacted to identify unpublished or ongoing clinical trials and to clarify data as required. Reference lists of the included studies were screened for relevant research. Finally, hand-searching was performed. The search strategy for PubMed is presented in Table 1.

Studies were selected independently and in duplicate by two authors (I.A.T., S.M.). Any inconsistencies were resolved by discussion with the other two authors (J.M.P., A.I.T.). They were not blinded while identifying the authors of the studies, their institutions, or their research findings. After the identification of potentially relevant studies by title, abstracts were read, and non-eligible studies were eliminated. After this stage, hand-searching of the references of the eligible studies was performed to find additional articles, which were not previously found. Finally, after reading the articles in full, the choice was made according to our inclusion and exclusion criteria (Table 2).

### 2.4. Data Items and Collection Extraction and Management

Two review authors (I.A.T., S.M.) performed data extraction independently and in duplicate. The information that was extracted included participants, intervention/appliance, treatment duration/observational period, outcomes, methods of outcome assessment, results, and conclusions. In case of no access to the missing data, only the existing data were reported and analyzed.

### 2.5. Risk of Bias/Quality Assessment in Individual Studies

The quality assessment of the included studies was performed using the ACROBAT-NRSI tool of Cochrane for non-randomized clinical trials and the Cochrane handbook for systematic reviews (chapter 8) for randomized clinical trials. Two review authors (I.A.T., S.M.) assessed the articles individually and then compared their findings. Any disagreements were resolved by discussion with the other two authors (J.M.P., A.I.T.). Regarding the randomized clinical trials, seven domains of bias were assessed: random sequence generation, allocation concealment, performance bias, detection bias, attrition bias, bias due to selective outcome reporting, and other sources of bias. A judgment of ‘low’, ‘high’, or ‘unclear’ risk of bias was made for each domain, while a final overall judgment was assessed based on the following:

1. Low risk of bias if all key domains of the study were at low risk of bias;
2. Unclear risk of bias if one or more key domains of the study were unclear;
3. High risk of bias if one or more key domains were at high risk of bias.

Concerning the non-randomized trials, bias due to confounding, bias in the selection of participants, bias in the measurement of interventions, bias due to departures from intended interventions, bias due to missing data, bias in the measurement of outcomes, and bias in the selection of the reported result were assessed for the qualitative evaluation of the study. Possible results for each domain and hence the overall evaluation of each study was: ‘low’, ‘moderate’, ‘serious’ risk of bias, and ‘no information’. We used the GRADE approach to interpret the results of this review.

### 2.6. Additional Analyses

Meta-analyses were undertaken using individual results on the change from the baseline in the parameters under study. They were summarized over all studies providing appropriate statistics (i.e., standard error/deviation for the change or *p*-value from a parametric test) using fixed or random-effects meta-analysis. Heterogeneity between studies was assessed using I2, and in the presence of significant heterogeneity, a random-effects model was used [18]. Stata command, metan, in Stata v13 was used for the analysis (StataCorp. 2013. Stata Statistical Software: Release 13. College Station, TX, USA: StataCorp LP).

## 3. Results

### 3.1. Study Selection and Characteristics

**Figure 1:**
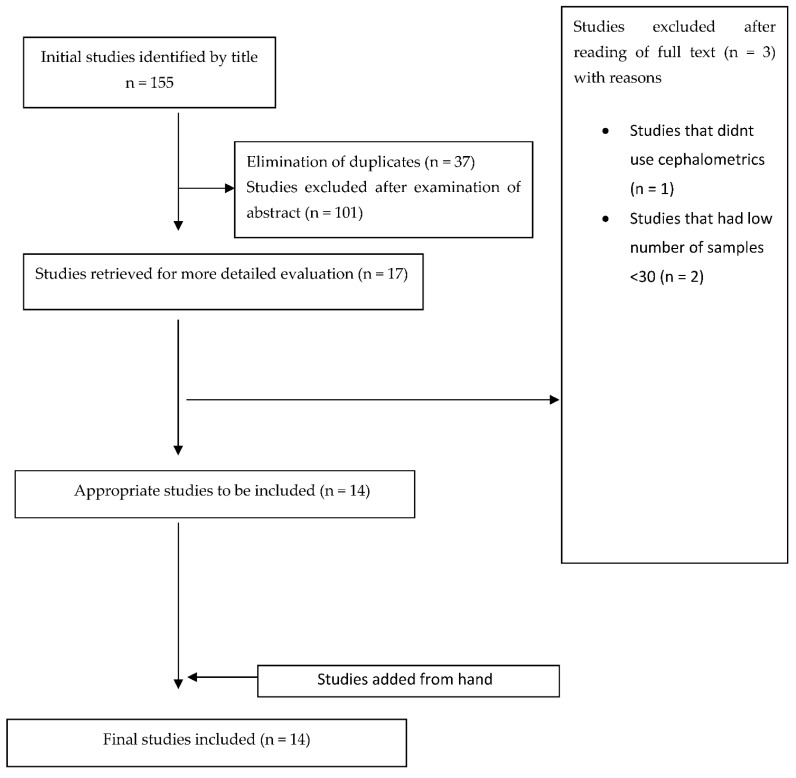
Flow diagram, selection of studies.

Our search resulted in 2297 articles. After title and abstract reading, irrelevant articles and duplicates were excluded, and 155 articles were read in full. Finally, 14 articles were selected for final analysis (two RCTs and 12 non-RCTs), based on our inclusion/exclusion criteria (Table 1) [19,20,21,22,23,24,25,26,27,28,29,30,31,32]. The procedure of article selection is presented on a flow diagram (Figure 1), and data are briefly presented in Table 3.

### 3.2. Risk of Bias within Studies

The seven criteria for the RCT bias assessment were random sequence generation, allocation concealment, performance bias, detection bias, attrition bias, selective reporting, and additional bias. One of the RCTs presented low risk of bias in all seven criteria, while the other study presented serious risk of bias due to random sequence generation, allocation concealment, attrition bias, and additional bias (Table 4).

The seven criteria for the non-RCT studies were: bias due to confounding, bias in the selection participants into the study, bias in the measurement of interventions, bias due to departures from intended interventions, bias due to missing data, bias in measurement outcomes and bias in the selection of the reported result. Seven studies presented a low risk of bias. None of the 12 studies showed bias in the selection of the reported result. One study presented a serious risk of bias due to confounding and in the measurement of intervention, while two studies presented a serious risk of bias due to confounding, in the selection of participants, in the measurement of intervention and the measurement of outcomes. Another study showed a serious risk of bias. Finally, one study presented bias in the selection of participants and the measurement of outcomes. Another study showed a serious risk of bias in five out of seven criteria. This study presented a serious risk of bias: due to confounding, in the selection of participants, in the measurement of intervention, due to missing data and in the measurement of outcomes (Table 5).

### 3.3. Results of Meta-Analysis

**Figure 2:**
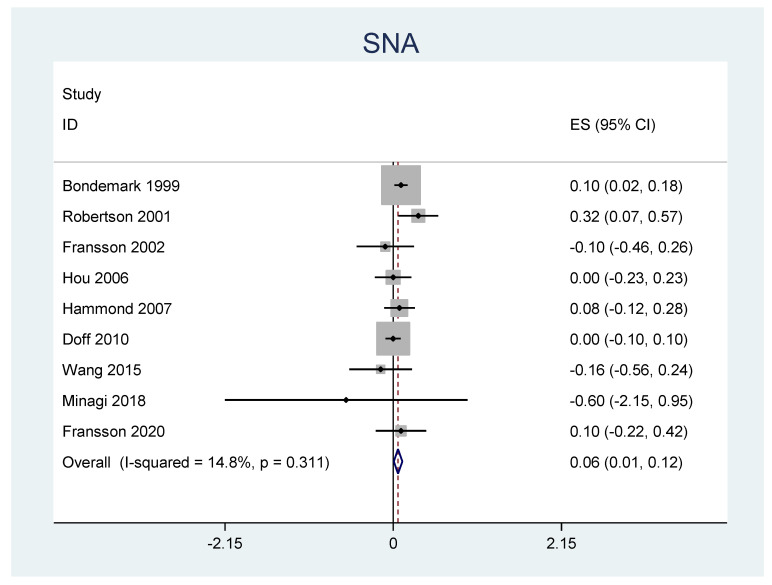
Forest plot of the results of SNA changes using the random-effects model.

**Figure 3:**
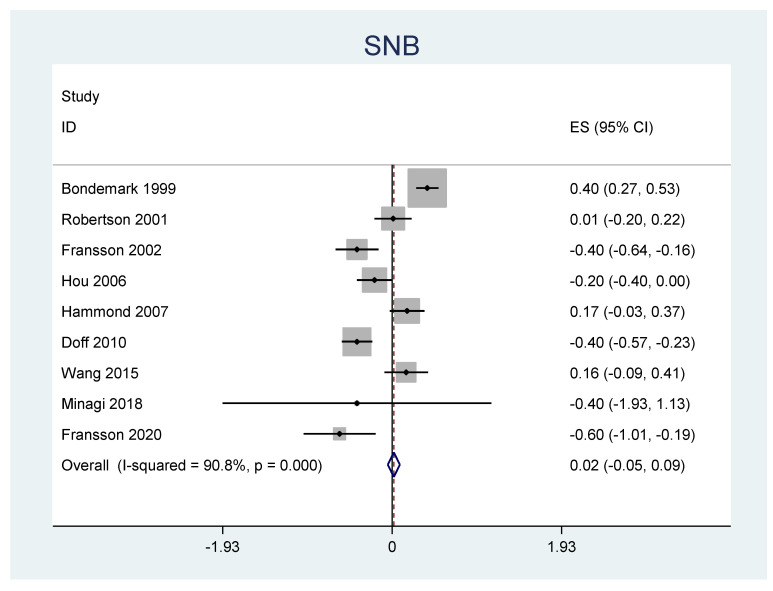
Forest plot of the results of SNB changes using the random-effects model.

**Figure 4:**
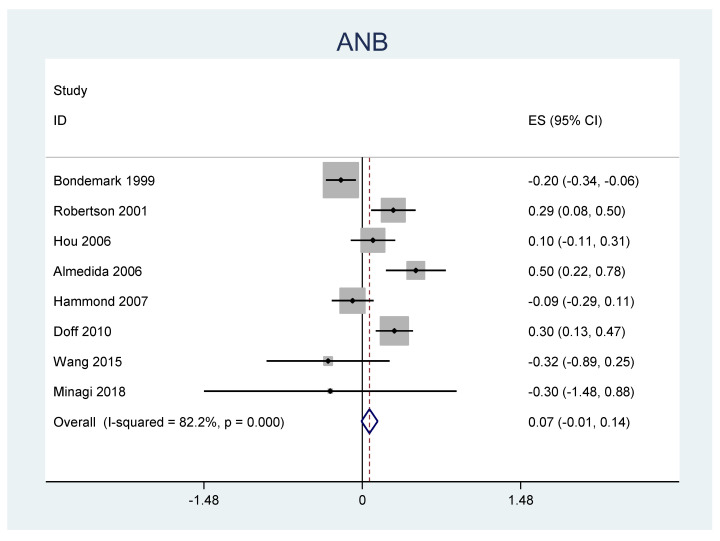
Forest plot of the results of ANB changes using the random-effects model.

**Figure 5:**
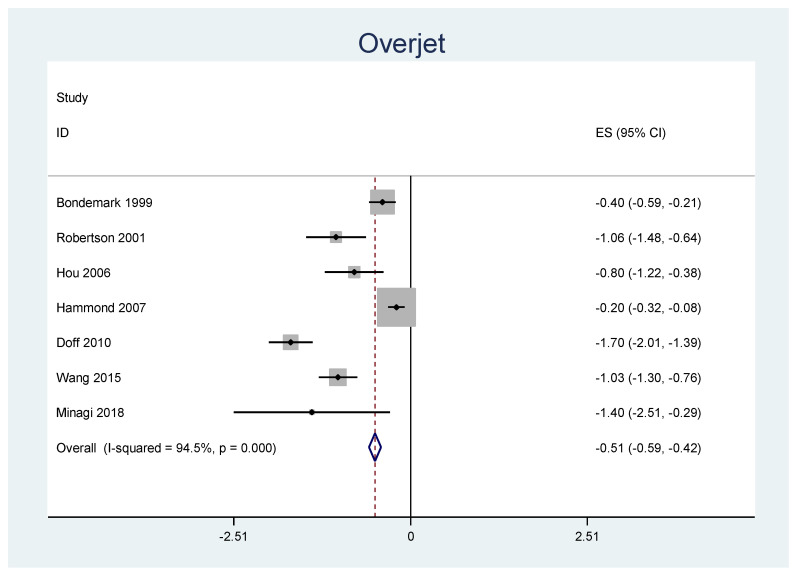
Forest plot of the results of overjet changes using the random-effects model.

**Figure 6:**
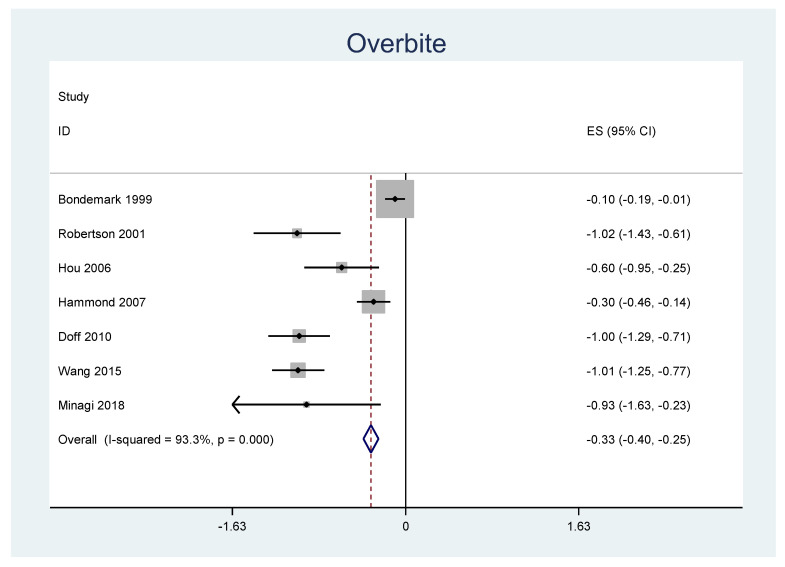
Forest plot of the results of overbite changes using the random-effects model.

**Figure 7:**
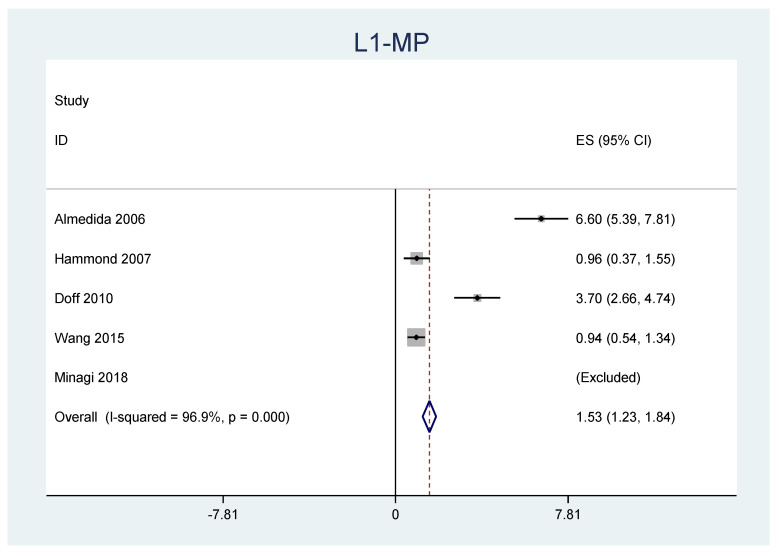
Forest plot of the results of L1-MP changes using the random-effects model.

A meta-analysis was performed for the outcomes of sella-nasion-pointA angle (SNA), sella-nasion-pointB angle (SNB), ANB angle, overjet, overbite, and L1-MP angle. (Table 6) There was no significant heterogeneity in SNA results (I2 = 14.8%, *p* = 0.31). An overall statistically significant positive change from baseline was found in SNA when studies were combined: 0.06 with 95% confidence interval (CI) (0.007, 0.116) (Figure 2). The SNB results were heterogeneous (I2 = 90.8%, *p* < 0.001). Overall estimated change from baseline in SNB was not statistically significant (*p* = 0.436): −0.099 with 95% CI (−0.347, 0.150) (Figure 3). Furthermore, the ANB results were heterogeneous (I2 = 82.2%, *p* < 0.001). Overall estimated change from baseline in ANB was not statistically significant (*p* = 0.360): 0.09 with 95% CI (−0.107, 0.296) (Figure 4). Overjet results were also heterogeneous (I2 = 94.5%, *p* < 0.001). An overall statistically significant decrease compared with baseline was found in overjet when studies were combined: −0.89 with 95% CI (−1.334, −0.459) (Figure 5). Overbite results were also heterogeneous (I2 = 93.3%, *p* < 0.001). An overall statistically significant decrease compared with baseline was found in overjet when studies were combined: −0.68 with 95% CI (−1.016, −0.344) (Figure 6). Finally, heterogeneity was found in L1-MP, between studies (I2 = 96.9%, *p* < 0.001). An overall positive and statistically significant change from baseline was found in L1-MP when studies were combined: 2.97 with 95% CI (0.993, 4.954) (Figure 7).

### 3.4. Dental Changes

Bondemark et al. found a reduction in overjet and overbite, but no significant changes in incisor inclination or interincisal angle [19]. A few years later, Rose et al. and Robertson et al. found that maxillary incisors were retroclined, mandibular incisors were proclined, and thus overjet and overbite were reduced [20,22,23]. Still, according to Rose et al., the interincisal angle was not altered [22]. Conversely, Fransson et al. observed a reduction in the interincisal angle, because only the lower incisors were proclined, without a significant inclination change in upper incisors [21]. On the other hand, in the RCT of Ringqvist et al., incisor inclination changed only vertically, without any further alteration in overjet, overbite, and interincisal angle [24].

In the following years, the studies of Almeida et al., Doff et al., and Wang et al. showed that both, proclination of lower incisors and retroclination of the upper incisors, led to overjet and overbite reduction [26,28,29]. Moreover, Hou et al. found a decrease in overjet and overbite without examining incisor inclination, and Hammond et al. observed only upper incisor posterior tipping [25,27]. Although the interincisal angle was reduced, according to Almeida et al., Doff et al., and Hammond et al., it did not change significantly in Wang et al. study [26,27,28,29].

More recently, Minagi et al. showed a significant decrease in overjet and overbite because of mandibular incisor proclination, while the interincisal angle remained unchanged [30]. Furthermore, Hamoda et al. found that both upper and lower incisors were tipped backward and forward, respectively [31]. Finally, Fransson et al. observed overjet and overbite reduction due to altered inclination of upper and lower incisors, after 10 years of mandibular protruding device use [32].

Regarding posterior teeth changes, Robertson et al. found that the upper first premolars and lower first molars had over-erupted while, Almeida et al. found that first upper and lowers molars, except from over-erupting, had also moved distally and mesially [23,26]. Moreover, Hammond et al. showed a mesial tip of the lower first molars, but without a change in the upper first molars [27].

### 3.5. Skeletal Changes

Regarding changes in mandibular position relative to the cranial base, in most studies, the mandibular plane angle was significantly increased, and thus the mandible had a more downward position [20,21,23,25,26,28,29,30]. Bondemark found a more downward and forward position and Ringqvist et al. a more downward and backward position [19,24]. On the other hand, Rose et al., Hammond et al., and Minagi et al. found no significant changes in mandibular position [22,27,30]. The vertical condylar position was also observed to be more downward by Robertson et al., but not in Almeida et al. study, in which there were no condylar changes [23,26]. Regarding the relation between the maxilla and the mandible and each jaw relationship with the cranial base, the ANB angle was found slightly reduced by Bondemark, because the SNB angle was increased, and the SNA angle did not change [19]. Robertson et al., also observed a decrease in ANB angle, because the SNA angle was reduced and SNB was not altered significantly [23]. On the other hand, the ANB angle was increased in the study by Hamoda et al., due to a decrease in the SNB angle and no significant change in the SNA angle [31]. Moreover, according to Doff et al., the ANB angle was increased, while the SNB angle was reduced, and the SNA angle did not change [28]. Conversely, some studies showed no significant change in the ANB angle [22,25,27,29,30]. Furthermore, the SNB angle was found slightly reduced by Fransson et al. [21,32].

Alterations in facial height were also observed in many studies. An increase in lower anterior facial height and thus an increase in total anterior facial height was found by most researchers, except Minagi et al. that found no significant change [19,21,23,24,25,26,28,29,30]. On the other hand, lower posterior facial height and total posterior facial height was found significantly increased only in two studies [23,25], while four other studies showed no significant change [19,21,28,29].

Bondemark also observed a significant increase in mandibular length [19]. On the contrary, a significant alteration in mandibular length was not found by others [21,24,25,26,27,28]. Although Fransson et al. did not find a significant change after 2 years of treatment, they observed a significant increase in patients that continued the treatment for 10 years as well as in patients that stopped the treatment [21,32].

The maxillary length was also increased in the studies by Robertson and Robertson et al. [20,23], but no significant change was observed in any other study [25,28,29].

Frasson et al. also observed an alteration in the hyoid bone position. More specifically, the distance between the hyoid bone and mandibular plane and between the hyoid bone and occlusal line was increased [21]. Moreover, a more downward hyoid bone position was observed by Fransson et al. after 10 years of oral appliance use, but it was also evident in patients that had stopped using the device [32].

## 4. Discussion

### Summary of Evidence

Different outcomes were searched among studies, the quality of which was also variable, according to the risk of bias assessment.

The two existing RCT studies were reported in 2003 by Ringvist et al. and in 2010 by Doff et al. [24,28]. The study of Ringvist et al. in 2003 presented a high risk of attrition bias [24]. In this study, patients that used two different methods of treatment at the same time were included in the final sample. Furthermore, this study presented a high risk of bias due to additional bias. In the study, a high number of patients did not attend the 4-year follow-up (15 in the MAD group and 6 in the uvulopalatopharyngoplasty -UPPP- group). On the other hand, the study of Doff et al. appeared to have a low risk of bias in all seven criteria for the outcome assessment [28]. Consequently, the statement that the use of MAD appliances leads to a decrease in overjet and overbite as well as results in shorter a downward and backward rotation of the mandible considered conclusions with a high level of evidence.

Twelve non-RCT studies were reported for the assessment of dental and skeletal effects from the long-term use of mandibular advancement devices. The study of Hou et al. showed a serious risk of bias due to confounding and in the measurement of intervention [25]. More specifically, the intervention status was not well-defined, and there were patients that switched treatment, which was not adjusted in the final analysis. The study of Almeida et al. presented a serious risk of bias due to confounding, in the selection of participants, in the measurement of intervention and the measurement of outcomes [26]. In this study, the start and follow-up measurements did not coincide, and the intervention status was not well-defined. The study of Hamoda et al. showed a serious risk of bias in those three criteria as well [31]. The most important bias in this study was the absence of the intervention status regarding the usage frequency of the devices. Hammond et al. showed a serious risk of bias in five out of seven criteria [27]. This study presented a serious risk of bias: due to confounding, in the selection of participants, in the measurement of intervention, due to missing data and in the measurement of outcomes. According to this study, there were patients that switched treatment, there were missing data, and the outcome assessor was aware of the intervention received by the participants (not blinded). The study by Wang et al. showed a serious risk of bias in the selection of participants and the measurement of outcomes [29]. The method of outcome assessment was subjective.

The remaining seven studies showed a low risk of bias in all criteria. Bondemark found that the use of MADs appliances resulted in decreased overjet and overbite, increased mandibular length, and the mandible moved forward and downwards [19]. Robertson in 2001 showed decreased overjet and overbite, increased lower incisor proclination, SNA, and ANB [20]. Robertson et al. in 2003 found that the use of MAD appliances resulted in over eruption of upper first premolars and lower first molars and proclination of the lower incisors [23]. Rose et al. showed an increase in incisor inclination, a mesial shift of the occlusion, and a decreased overjet and overbite [22]. Fransson et al. in 2002 found an increase in the posterior rotation of the mandible as long as an increased proclination of the mandibular incisors [21]. The study of Minagi et al. resulted in decreased overjet and overbite [30]. In 2020 Fransson et al. concluded that the use of MADs resulted in proclination of the mandibular incisors with consequent decreased overjet and overbite [32]. All these statements are considered to be strong since those researches had a low risk of bias.

The heterogeneity was high for all the outcomes in this meta-analysis. A possible explanation for this might be the different treatment duration among the included studies. The mean treatment duration was less or equal to 3 years in most studies [19,20,21,22,23,25,27,28], 4 years for two studies [29,30], 7 years in one study [26], and over 10 years in two studies [31,32].

Moreover, the design of the mandibular advancement devices was different in most of the studies [19,20,21,22,23,24,25,26,27,28,29,30,31,32]. All the oral appliances protruded the mandible and provided full occlusal coverage [19,20,21,22,23,25,26,27,28,29,30,31,32], except for one that provided occlusal coverage only for the posterior teeth [24]. There was also variation in the amount of protrusion for each oral appliance. A 65–75% of the maximum protrusion with a small vertical opening was used in most studies [19,20,21,22,23,25,26,27,28,29,30,31,32], but 50% of the maximum protrusion was chosen by one study [24]. In the study by Ringvist et al. that used the appliance that provided only posterior occlusal coverage and protruded the mandible, only 50% showed no change in overjet and overbite. On the other hand, a systematic review and meta-analysis found no significant difference between the side effects caused by 50% and 75% of maximum protrusion [33].

Furthermore, adherence to the oral appliance daily wear might be a factor that increased outcome heterogeneity. All studies required the patients to wear the oral appliances at least 5 h a day and at least 4 days per week. The non-adherent patients were also excluded from all studies. Nevertheless, no objective method was used to record patient total wear time and thus evaluate true patient compliance.

Finally, one of the outcomes that was controversial is the extrusion of the molars as an effect of the MADs use. An explanation on that disagreement between the studies can be that MADs design differs on the occlusal coverage. More specific, the studies that used appliances with occlusal coverage could prevent dental extrusion while the ones that do not have the coverage could not. In the near future, 3D printing technology can be helpful in order to fabricate those appliances and possibly be delivered the same day to the patient’s mouth. There are lots of other appliances in the field of orthodontics that have been fabricated with 3D printing technology and proved to be significantly successful for the treatment outcome [34]. The limitation of this study is the small number of randomized clinical trials in the present literature.

## 5. Conclusions

Regarding dental and skeletal side effects caused by mandibular advancement appliances used for adult OSA treatment, the current level of evidence is weak. The meta-analysis results suggest that mandibular advancement devices used for OSA treatment increase the lower incisor proclination by 1.54 ± 0.16°, decrease overjet by 0.89 ± 0.04 mm, decrease overbite by 0.68 ± 0.04 mm, rotate the mandible downward and forward, and increase SNA angle by to 0.06 ± 0.03°. Some of those results are clearly not clinically significant. More randomized clinical trials providing high-quality evidence are needed.

## Figures and Tables

**Table 1:** The search strategy for PubMed. Abbreviations: Mesh—Medical Subject Headings.

((“Sleep Apnea Syndromes”(Mesh)) AND “adverse effects” (Subheading))	2163 results
(((“Sleep Apnea Syndromes” (Mesh)) AND “adverse effects” (Subheading)) AND “Jaw” (Mesh))	94 results
((“Sleep Apnea Syndromes” (Mesh)) AND “Jaw” (Mesh) AND “Tooth” (Mesh))	33 results
((((“Sleep Apnea Syndromes” (Mesh)) AND “adverse effects” (Subheading)) AND “Jaw” (Mesh)) AND “Tooth” (Mesh))	7 results

**Table 2:** Inclusion and exclusion criteria.

Inclusion Criteria	Exclusion Criteria
Studies that refer to oral appliance use for the treatment of OSA/snoring and its side effects in occlusion and skeletal tissues.	Studies that refer to non-specific side effects of oral appliance use or treatment of OSA/snoring, such as tooth discomfort and increased salivation.
RCTs, non-randomized trials (prospective or retrospective).	Studies that refer to side effects of oral appliance use for other reasons, than to treat OSA/snoring.
Studies in humans.	Case reports, case series, reviews, guidelines, and authors’ opinion.
Studies in adults with sufficient number of teeth to retain the oral appliance.	

**Table 3:** Data extraction.

Authors/Publication Year	Study Design	Participants (Number-Age-Gender-AHI)	Intervention/Appliance	Treatment Duration/Observational Period	Outcomes	Method of Outcome Assessment	Results	Conclusion
Bondemark [19] (1999)	Prospective	30 obstructive sleep apnea (OSA)/snoring patients (21 males (M), 9 females (F), mean age 55.3 ± 8.61 months)	Monobloc acrylic mandibular advancement splint, with 8 posterior stainless steel caps and full tooth coverage	2 years (y)	Sagittal and vertical, dental, and skeletal measurements Mandibular length measurements Angle measurements	Baseline and follow-up cephalometric radiographs	Decreased overjet (OJ) and overbite (OB) Increased sella-nasion-pointB angle (SNB), mandibular plane to cranial base angle (ML/NSL) and decreased pointA-nasion-pointB angle (ANB) Increased mandibular length and more forward and downward mandibular position Forward mandibular movement correlated with mandibular length change and SNB	Forward and downward change in mandibular position, due to increase in mandibular length
Robertson [20] (2001)	Prospective	100 OSA/snoring patients (87M, 13F, mean age 49 ± 8.5 years)	Non-adjustable mandibular advancement splint with full tooth coverage	6–30 months (6 months intervals)	Dentoalveolar and skeletal measurements	Baseline and follow-up cephalometric radiographs	Increased sella-nasion-pointA angle (SNA), ANB, anterior nasal spine to posterior nasal spine distance (ANS-PNS), vertical condylar position relative to cranial base (Cd-vert), lower and total anterior and posterior facial height Decrease in OJ and OB Decreased angle of upper incisor axis to anterior nasal spine/posterior nasal spine line (Ui/ANS-PNS) (palatal tipping) and increased angle of lower incisor axis to mandibular plane (Li/Me-Go) (labial tipping) Changes over time	Mainly minor skeletal and dental changes
Fransson et al. [21]. (2002)	Prospective	65 patients (52M, 13F, mean age 54.8 ± 9.0 years, 44 OSA, 21 snoring)	Monobloc heat-cured methyl methacrylate mandibular protruding device with 4 metal caps for molars and full tooth coverage	2 years	Dentofacial measurements Pharyngeal measurements	Baseline and follow-up cephalometric radiographs	Increased cranial base to occlusal plane SN/OL, anterior facial height and decreased SNB Increased lower incisor axis to mandibular line angle (ILi/ML) (proclination of lower incisors) Increased distance between the hyoid bone, maxilla (hy-NL) and mandible (hy-ML)	Posterior rotation of the mandible and proclination of mandibular incisors
Rose et al. [22] (2002)	Retrospective	34 mild–moderate OSA patients (mean age 52.9 ± 9.6 years, mean body mass index (BMI) 28.6 ± 4.2 kg/m^2^)	Mandibular advancement device (MAD) consisted of 2 hard acrylic plates joined by U-shaped clasps (Karwwetzky U-clasp activator)	29.6 ± 5.1 months	Dentofacial cephalometric measurements Dental cast analysis	Baseline and follow-up dental casts and cephalometric radiographs	OJ and OB decrease Is-SN decrease (retroclination of upper incisors) Ii-Me-Go increase (proclination of lower incisors) Dental cast analysis Decrease in OJ, OB, posterior OB (bilaterally), molar relationship (bilaterally) Increase in anterior arch length and overlaps/spaces reduction	Incisor inclination and mesial shift of the occlusion
Robertson et al. [23] (2003)	Longitudinal, observational study	100 OSA/snoring patients (87M, 13F, mean age 49 ± 8.5 years)	Non-adjustable mandibular advancement splint ith full tooth coverage	6–30 months (6 months intervals)	Dentoalveolar skeletal measurements	Baseline and follow-up cephalometric radiographs	Combined group: increase in SNA, ANB, anterior facial height, posterior and mainly in lower facial height, maxillary length, vertical mandibular position. Mandibular first molars and maxillary first premolars overeruption, retroclination of upper incisors and proclination of lower incisors, reduction in OJ, OB and maxillary arch length. Positive correlation between device advancement and ANB angle. 6 months: facial height increase, downward mandibular position, OJ and OB decrease 12 months: vertical mandibular position increase 18 months: total and lower facial height increase, vertical mandibular position, OJ and OB decrease 24 months: increase in facial height, SNA, vertical mandibular position. Over-eruption of mandibular first molars and maxillary premolars and proclination of mandibular incisors 30 months: proclination of mandibular incisors, OJ and OB decrease. Reduced lower facial height compared with 18 and 24 months. Positive correlation of MAD anterior opening and OB change.	Changes in facial height, overjet, overbite, and position of the mandible even before 6 months of device use. Over-eruption of upper first premolars and lower first molars and proclination of lower incisors occurred after 2 years of device use. Overbite changes might be decreased by keeping a minimum bite opening
Ringqvist [24] (2003)	Randomized clinical trial	45 OSA patients treated with MAD (mean age 48.9 years, mean weight 87.8 kg, mean BMI 27.0 kg/m^2^) 43 OSA patients treated with uvulopalatopharungoplasty (UPPP): mean age 51 years, mean weight 87.8 kg, mean BMI 27.1 kg/m^2^	MAD was a mono-bloc device consisted of heat-cured methyl methacrylate.	MAD patients (4.1 years, 30 patients completed the follow-up and 27 were only treated with MAD) UPPP patients (4.3 years, 37 completed the follow-up and 27 were only treated with UPPP)	O1: MAD group dental and skeletal measurements O2: UPPP group dental and skeletal measurements	Lateral cephalometric radiographs with the patient in supine position.	O1: Significant alterations in horizontal (Is-NSL) and vertical upper incisor position (Is-ML), and in horizontal position of lower incisors (Ii-NSL) No significant changes in overjet, overbite, and mandibular length Significant change in horizontal position (B-B’) and inclination of the mandible (ML-NSL) Increase in the Is-NSL, Is-ML and Ii-NSL distances was correlated with an increased angle ML/NSL O2: Significant increase in Ii-NSL	Minor dental and skeletal changes after 4 years of MAD use. No clinically important differences between MAD and UPPP groups
Hou et al. [25] (2006)	Prospective	67 Chinese OSA patients (50M, 17F, mean age 46.9 ± 8.9 years)	modified Harvold monobloc type of functional appliance	1–3 years 1 year: *n* = 63 2 years: *n* = 43 3 years: *n* = 30	Dental and skeletal measurements	Baseline and follow-up cephalometric radiographs	Increased mandibular plane to cranial base angle (MnPl/SN) Increased lower (LFH) and total anterior and posterior facial height (TFH) Decreased OJ and OB Changes over time	Small dentofacial changes and main OJ and OB reduction during early treatment
Almeida et al. [26] (2006)	Retrospective	71 OSA patients (63M, 8F, mean age 49.7 ± 9.7 years, respiratory disturbance index 28.9 ± 17.0/h, BMI 29.3 ± 5.9 kg/m^2^)	Klearway oral appliance	7.3 ± 2.1 years on average	Dental, skeletal, and upper airway measurements Changes over time	Baseline and follow-up cephalometric radiographs	Decreased upper incisor (U1-SN and U1-PP) and upper molar inclination (U6-SN and U6-PP), upper to lower molar distance projected to cranial base (U6-L6-SN), OJ, and OB Increased L1-MP, lower molar to mandibular plane angle (L6-MP), cranial base to mandibular plane angle (SN-MP) and palatal plane to mandibular plane angle (PP-MP), maxillary molar height (MXMH) and mandibular molar height (MDMH), ANB, LFH and TFH Changes according to baseline Angle classification Changes according to baseline OB Correlations	Craniofacial and dental changes occur after long-term OA use
Hammond et al. [27] (2007)	Retrospective	64 OSA patients (50M, 14F)	2-piece acrylic appliance with full occlusal coverage and a screw that titrates the device (Mehta et al.)	25.1 ± 11.8 months on average	Dental, skeletal, and anthropometric measurements Subjective side effects and satisfaction with the oral appliance	Baseline and follow-up cephalometric radiographs, study model analysis and anthropometric measurements Questionnaire	Cephalometric analysis on 46 patients (34M, 12F): Sagittal changes: vertical upper incisor position (ii-OLp: mean 0.52 mm), vertical lower incisor position (mi-OLP: mean 0.26 mm) Increased upper incisor to cranial base angle (ii/MP: mean 0.96°) Decreased interincisal angle (ii/is: mean −1.69°), and upper incisor to occlusal plane angle (ii/OL: mean −1.02°)	Minor dental and skeletal side effects
Doff et al. [28] (2010)	Randomized clinical trial	103 OSA patients (51 with MAD)	Thorton Adjustable positioner	2.3 ± 0.2 years on average	Dental and skeletal measurements	Baseline and follow-up cephalometric radiographs	Decreased OJ, OB, SNB, upper incisor to palatal plane angle, interincisal angle, and anterior facial height ratio Increased ANB, lower incisor to mandibular plane angle, LFH and TFH Downward and backward rotation of the mandible Decreased shortest linear distance menton line SN-perpendicular (Me-hor) and increased shortest linear distance menton line SN	Mainly dental changes
Wang et al. [29] (2015)	Prospective	42 patients OSA patients (31M, 11F, mean age 47 ± 10 years, mean AHI 27 ± 19)	Silensor appliance	4 ± 3 years on average	Dental and skeletal measurements Changes over time Subjective side effects	Questionnaire and baseline and follow-up cephalometric radiographs	Decreased OJ, OB, U1-SN and upper incisor axis to nasion-pointA line (U1-NA) angle, U1-NA distance Increased L1-MP and lower incisor to nasion-pointB line (L1-NB) angle, mandibular plane to Franfort horizontal plane, anterior LFH and TFH Changes prior to and over 3 years of treatment Reduction in most subjective side effects at follow-up	Minor dental and skeletal side effects (1–3 years of treatment mainly skeletal changes, after 3 years of treatment dental and skeletal changes)
Minagi et al. [30] (2018)	Retrospective	64 OSA patients (44M, 20F, mean age 57.7 ± 14.2 years, mean BMI 23.9 ± 3.6 kg/m^2^, mean apnea-hypopnea index (AHI) 24.9 ± 14.7	Mad consisted of two separate acrylic monoblock modified plates (ERKODRNT)	4.3 ± 2.1 years on average	Dental and skeletal measurements Rate of changes Predictors of changes	Baseline and follow-up cephalometric radiographs	Decreased OJ, OB and L1-MP angle Great OJ decrease (≥1 mm) correlated with treatment duration, MAD use frequency and mandibular advancement rate. Weak negative correlation between total number of teeth and decrease in OJ Weak negative correlation between maxillary teeth and decrease in OJ	Dental side effects (low number of maxillary teeth and MAD treatment duration, use frequency and mandibular advancement correlated with OJ reduction)
Hamoda et al. [31] (2018)	Retrospective	62 patients with primary snoring or mild to severe OSA (52M, 10F, mean age 49 ± 8.6 years, mean BMI 29.1 ± 6.9 kg/m^2^, mean AHI 30.0 ± 14.6 for 56 patients, Angle Class I/Class II/Class III 31/26/4)	Klearway^®^ or SomnoDent^®^	12.6 ± 3.9 years on average	Dental and skeletal measurements Rate of changes Predictors of changes	Baseline and follow-up cephalometric radiographs (up to 9 cephalometric radiographs for some patients)	Decreased OJ, OB and L1-MP angle Greater OJ decrease (≥1 mm) correlated with treatment duration, MAD use frequency and mandibular advancement rate. Upper incisor retroclination (U1-SN, U1-PP, U1-NA) with constant rate over the years (U1-SN reduction of 0.49°/year) Lower incisor proclination (L1-NB, L1-MP) with declining and not constant rate over the years Minor posterior and downward mandibular movement (decrease: in SNB 0.7° with a constant rate of 0.05°/year and mean ANB reduction of 0.43° and mean increase in: mandibular plane to Frankfort horizontal plane angle (MPFH) 1.1° and in cranial base to gonion-gnathion line angle (SNGoGn) 0.9°) Treatment duration correlated with all the cephalometric variables that changed Greater baseline BMI correlated with greater upper incisor retroclination and higher baseline ANB angle with greater mandibular incisor proclination	Dental changes happen progressively and duration of mandibular advancement device treatment is the greatest factor of their magnitude Minor skeletal changes that occur are not clinically significant
Fransson et al. [32] (2020)	Prospective	65 patients (52M, 13F, mean age 54.8 ± 9.0 years, 44 OSA, 21 snoring)	Monobloc heat-cured methyl methacrylate mandibular protruding device with 4 metal caps for molars and full tooth coverage	10 years	Dentofacial measurements Pharyngeal measurements	Baseline and follow-up cephalometric radiographs	Increased SN/OL, SN/ML, anterior facial height and decreased SNB Increased ILi/ML (proclination of lower incisors) Increased distance between the hyoid bone, maxilla (hy-NL) and mandible (hy-ML)	Posterior rotation of the mandible and proclination of mandibular incisors

**Table 4:** Risk of bias assessment for randomized clinical trials. Abbreviations: CPAP—continuous positive airway pressure, MAD(s)—mandibular advancement device(s), UPPP—uvulopalatopharyngoplasty.

Author (Year)	Outcomes	Random Sequence Generation	Allocation Concealment	Performance Bias	Detection Bias	Attrition Bias	Selective Reporting	Other	Overall
Ringqvist et al. [24] (2003)	O1: dental and skeletal measurements on MAD patients O2: dental and skeletal measurements on UPPP patients	Unclear for all outcomes (‘…45 were randomly assigned to treatment with the mandibular advancement device (MAD) group and 43 to treatment with UPPP’) not possible to conclude if randomization was successful	Unclear for all outcomes (not mentioned concealment of allocation, probably not performed)	Low for all outcomes (not mentioned blinding of participants/personnel but the outcome is not likely to be affected)	Unclear for all outcomes (blinding of outcome assessor is not mentioned)	High for all outcomes (patients that did not attend the 4-year follow-up were 15 in the MAD group and 6 in the UPPP group)	Low for all outcomes (all pre-specified variables were measured)	High for all outcomes (patients received both treatments, 3 patients in the MAD group and 10 in the UPPP group)	High for all outcomes (patients not attending the follow-up and patients receiving both treatments can affect the outcomes)
Doff et al. [28] (2010)	Craniofacial changes	Unclear for all outcomes (‘…patients were randomized’) not possible to conclude if randomization was successful	Unclear for all outcomes (not mentioned concealment of allocation, probably not performed)	Low for all outcomes (not mentioned blinding of participants/personnel but the outcome is not likely to be affected)	Low for all outcomes (‘…one blinded observer (MD) performed all tracings’)	Low for all outcomes (number of missing outcome data balanced among groups-reasons not related to outcome)	Low for all outcomes (all pre-specified variables were measured)	Low for all outcomes (patients that randomized in oral appliance treatment, and after treated for 3 months, changed to CPAP treatment were excluded)	Low for all outcomes (no concealed allocation but baseline characteristics that can affect the outcome -AHI, BMI, number of teeth, appliance usage, were similar among groups)

**Table 5:** Risk of bias assessment for non-randomized controlled trials.

Author (Year)	Outcomes	Bias Due to Confounding	Bias in Selection of Participants into the Study	Bias in Measurement of Interventions	Bias Due to Departures from Intended Interventions	Bias Due to Missing Data	Bias in Measurement of Outcomes	Bias in Selection of the Reported Result	Overall Bias
Bondemark [19] (1999)	Mandibular and dentofacial changes	Low for all outcomes	Low for all outcomes (all eligible participants were included and start of intervention and follow-up coincide)	Low for all outcomes (well-defined intervention status)	Low for all outcomes (no bias due to departure from intervention is expected)	Low for all outcomes (data were reasonably complete)	Low for all outcomes (objective method of outcome assessment, any error is unrelated to intervention status)	Low for all outcomes (all reported results correspond to intended outcome)	Low for all outcomes
Robertson [20] (2001)	Dentoalveolar and skeletal changes	Low for all outcomes	Low for all outcomes	Low for all outcomes	Low for all outcomes	Low for all outcomes (no missing outcome data)	Low for all outcomes (all pre-specified variables were measured)	Low for all outcomes (no possible risk of bias from other source)	Low for all outcomes
Robertson et al. [23] (2003)	Dentoalveolar and skeletal changes	Low for all outcomes	Low for all outcomes	Low for all outcomes	Low for all outcomes	Low for all outcomes (no missing outcome data)	Low for all outcomes (all pre-specified variables were measured)	Low for all outcomes (no possible risk of bias from other source)	Low for all outcomes
Rose et al. [22] (2002)	Dentofacial cephalometric and dental casts measurements	Low for all outcomes	Low for all outcomes	Low for all outcomes	Low for all outcomes	Low for all outcomes	Low for all outcomes	Low for all outcomes	Low for all outcomes
Fransson et al. [21] (2002)	O_1_: airway changes O_2_: skeletal, dental, soft tissue changes	Low for all outcomes	Low for all outcomes (all eligible participants were included and start of intervention and follow-up coincide)	Serious for all outcomes (intervention status regarding usage frequency not well-defined)	Low for all outcomes (no bias due to departure from intervention is expected)	Low for all outcomes (data were reasonably complete)	Low for all outcomes (objective method of outcome assessment, any error is unrelated to intervention status, outcome assessor was blinded during cephalometric analysis.)	Low for all outcomes (all reported results correspond to intended outcome)	Low for all outcomes
Hou et al. [25] (2006)	Long-term dentofacial changes	Serious for all outcomes (at least one critically important domain not appropriately measured or not adjusted for)	Low for all outcomes (all eligible participants were included and start of intervention and follow-up coincide)	Serious for all outcomes (intervention status not well-defined)	Serious for all outcomes (switches in treatment is apparent and are not adjusted in for the analysis)	Low for all outcomes (data were reasonably complete)	Low for all outcomes (objective method of outcome assessment, any error is unrelated to intervention status)	Low for all outcomes (all reported results correspond to intended outcome)	Serious for all outcomes (the study is judged to be in serious risk of bias in at least one domain)
Almeida et al. [26] (2006)	Skeletal, dental, and occlusal changes	Serious for all outcomes (at least one critically important domain not appropriately measured or not adjusted for)	Serious for all outcomes (retrospective study (start follow-up did not coincide) selection into the study was related to intervention and possibly to outcome)	Serious for all outcomes (intervention status not well-defined)	Low for all outcomes (no bias due to departure from intervention is expected)	Low for all outcomes (data were reasonably complete)	Serious for all outcomes (outcome assessor was aware of the intervention received by the participants)	Low for all outcomes (all reported results correspond to intended outcome)	Serious for all outcomes (the study is judged to be in serious risk of bias in at least one domain)
Hammond et al. [27] (2007)	O_1_: long-term subjective side-effects O_2_: long-term dental and skeletal effects side-effects	Serious for all outcomes (at least one critically important domain not appropriately measured or not adjusted for)	Serious for all outcomes (inception bias)	Serious for all outcomes (intervention status not well-defined)	Low for O_1_ outcomes Serious for O_2_ outcomes (switches in treatment)	Serious for all outcomes (missing data-baseline characteristics; the risk of bias cannot be removed trough appropriate analysis)	Serious for O_1_ (subjective method of outcome assessment) Serious for O_2_ (outcome assessor was aware of the intervention received by the participants)	Low for all outcomes (all reported results correspond to intended outcome)	Serious for all outcomes (the study is judged to be in serious risk of bias in at least one domain)
Wang et al. [29] (2015)	O_1_: long-term subjective side-effects O_2_: long-term dental and skeletal effects side-effects	Serious for all outcomes (at least one critically important domain not appropriately measured or not adjusted for)	Low for all outcomes (all eligible participants were included and start of intervention and follow-up coincide)	Low for all outcomes (well-defined intervention status)	Low for all outcomes (no bias due to departure from intervention is expected)	Low for all outcomes (data were reasonably complete)	Serious for O_1_ outcome (subjective method of outcome assessment) Low for O_2_ outcomes (objective method of outcome assessment, any error is unrelated to intervention status, outcome assessor was blinded during cephalometric analysis.)	Low for all outcomes (all reported results correspond to intended outcome)	Serious for all outcomes (the study is judged to be in serious risk of bias in at least one domain)
Minagi et al. [30] (2018)	causing factors and predictors of orthodontic changes after long-term use	Low for all outcomes	Low for all outcomes (all eligible participants were included and start of intervention and follow-up coincide)	Low for all outcomes (well-defined intervention status)	Low for all outcomes (no bias due to departure from intervention is expected)	Low for all outcomes (data were reasonably complete)	Low for all outcomes (objective method of outcome assessment, any error is unrelated to intervention status, outcome assessor was blinded during cephalometric analysis.)	Low for all outcomes (all reported results correspond to intended outcome)	Low for all outcomes
Hamoda et al. [31] (2018)	O1: dental and skeletal changes O2: Rate and predictors of changes	Serious for all outcomes (at least one critically important domain not appropriately measured or not adjusted for)	Serious for all outcomes (retrospective study)	Serious for all outcomes (intervention status regarding usage frequency not well-defined)	Low for all outcomes (no bias due to departure from intervention is expected)	Low for all outcomes (data were reasonably complete)	Low for all outcomes (objective method of outcome assessment)	Low for all outcomes (all reported results correspond to intended outcome)	Serious for all outcomes (the study is judged to be in serious risk of bias in at least one domain)
Fransson et al. [32] (2020)	O_1_: airway changes O_2_: skeletal, dental, soft tissue changes	Serious for all outcomes (at least one critically important domain not appropriately measured or not adjusted for)	Low for all outcomes (all eligible participants were included and start of intervention and follow-up coincide)	Low for all outcomes (well-defined intervention status)	Low for all outcomes (no bias due to departure from intervention is expected)	Low for all outcomes (data were reasonably complete)	Low for all outcomes (objective method of outcome assessment, any error is unrelated to intervention status, outcome assessor was blinded during cephalometric analysis.)	Low for all outcomes (all reported results correspond to intended outcome)	Low for all outcomes

**Table 6:** Overall results of meta-analysis. Mean difference, upper limit, and standard deviation.

Parameters	ES (Mean Diff.)	Upper Limit	SD
SNA	0.061	0.116	0.028
SNB	0.019	0.088	0.035
ANB	0.067	0.143	0.039
Overjet	−0.506	−0.420	0.044
Overbite	−0.326	−0.255	0.036
L1-MP	1.535	1.838	0.155

## Data Availability

Not applicable.

## References

[1] Sateia M.J. (2014). International classification of sleep disorders-third edition: Highlights and modifications. Chest.

[2] Koutsourelakis I., Perraki E., Zakynthinos G., Minaritzoglou A., Vagiakis E., Zakynthinos S. (2012). Clinical and polysomnographic determinants of snoring. J. Sleep Res..

[3] Tsolakis I.A., Venkat D., Hans M.G., Alonso A., Palomo J.M. (2016). When static meets dynamic: Comparing cone-beam computed tomography and acoustic reflection for upper airway analysis. Am. J. Orthod Dentofac. Orthop..

[4] Rohra A.K., Demko C.A., Hans M.G., Rosen C., Palomo J.M. (2018). Sleep disordered breathing in children seeking orthodontic care. Am. J. Orthod Dentofac. Orthop..

[5] Caples S.M., Gami A.S., Somers V.K. (2005). Obstructive sleep apnea. Ann. Intern. Med..

[6] Behrents R.G., Shelgikar A.V., Conley R.S., Flores-Mir C., Hans M., Levine M., McNamara J.A., Palomo J.M., Pliska B., Stockstill J.W. (2019). Obstructive sleep apnea and orthodontics: An American Association of Orthodontists White Paper. Am. J. Orthod Dentofac. Orthop..

[7] Punjabi N.M. (2008). The epidemiology of adult obstructive sleep apnea. Proc. Am. Thorac. Soc..

[8] Williams S.K., Ravenell J., Jean-Louis G., Zizi F., Underberg J.A., McFarlane S.I., Ogedegbe G. (2011). Resistant hypertension and sleep apnea: Pathophysiologic insights and strategic management. Curr. Diab. Rep..

[9] Usmani Z.A., Chai-Coetzer C.L., Antic N.A., McEvoy R.D. (2013). Obstructive sleep apnoea in adults. Postgrad. Med. J..

[10] Ramar K., Dort L.C., Katz S.G., Lettieri C.J., Harrod C.G., Thomas S.M., Chervin R.D. (2015). Clinical Practice Guideline for the Treatment of Obstructive Sleep Apnea and Snoring with Oral Appliance Therapy: An Update for 2015. J. Clin. Sleep Med..

[11] Marklund M., Braem M.J.A., Verbraecken J. (2019). Update on oral appliance therapy. Eur. Respir. Rev..

[12] Chen A., Burger M.S., Rietdijk-Smulders M.A.W.J., Smeenk F.W.J.M. (2020). Mandibular advancement device: Effectiveness and dental side effects. A real-life study. Cranio.

[13] Hoekema A., Stegenga B., De Bont L.G. (2004). Efficacy and co-morbidity of oral appliances in the treatment of obstructive sleep apnea-hypopnea: A systematic review. Crit Rev. Oral Biol. Med..

[14] Araie T., Okuno K., Ono Minagi H., Sakai T. (2018). Dental and skeletal changes associated with long-term oral appliance use for obstructive sleep apnea: A systematic review and meta-analysis. Sleep Med. Rev..

[15] Patel S., Rinchuse D., Zullo T., Wadhwa R. (2019). Long-term dental and skeletal effects of mandibular advancement devices in adults with obstructive sleep apnoea: A systematic review. Int. Orthod..

[16] Bartolucci M.L., Bortolotti F., Martina S., Corazza G., Michelotti A., Alessandri-Bonetti G. (2019). Dental and skeletal long-term side effects of mandibular advancement devices in obstructive sleep apnea patients: A systematic review with meta-regression analysis. Eur. J. Orthod..

[17] Martins O.F.M., Chaves Junior C.M., Rossi R.R.P., Cunali P.A., Dal-Fabbro C., Bittencourt L. (2018). Side effects of mandibular advancement splints for the treatment of snoring and obstructive sleep apnea: A systematic review. Dent. Press J. Orthod..

[18] DerSimonian R., Laird N. (1986). Meta-analysis in clinical trials. Control Clin. Trials.

[19] Bondemark L. (1999). Does 2 years’ nocturnal treatment with a mandibular advancement splint in adult patients with snoring and OSAS cause a change in the posture of the mandible?. Am. J. Orthod. Dentofac. Orthop..

[20] Robertson C.J. (2001). Dental and skeletal changes associated with long-term mandibular advancement. Sleep.

[21] Fransson A.M., Tegelberg A., Svenson B.A., Lennartsson B., Isacsson G. (2002). Influence of mandibular protruding device on airway passages and dentofacial characteristics in obstructive sleep apnea and snoring. Am. J. Orthod. Dentofac. Orthop..

[22] Rose E.C., Staats R., Virchow C., Jonas I.E. (2002). Occlusal and skeletal effects of an oral appliance in the treatment of obstructive sleep apnea. Chest.

[23] Robertson C., Herbison P., Harkness M. (2003). Dental and occlusal changes during mandibular advancement splint therapy in sleep disordered patients. Eur. J. Orthod..

[24] Ringqvist M., Walker-Engström M.L., Tegelberg A., Ringqvist I. (2003). Dental and skeletal changes after 4 years of obstructive sleep apnea treatment with a mandibular advancement device: A prospective, randomized study. Am. J. Orthod. Dentofac. Orthop..

[25] Hou H.M., Sam K., Hägg U., Rabie A.B.M., Bendeus M., Yam L.Y.C., Ip M.S. (2006). Long-term dentofacial changes in Chinese obstructive sleep apnea patients after treatment with a mandibular advancement device. Angle Orthod..

[26] Almeida F.R., Lowe A.A., Sung J.O., Tsuiki S., Otsuka R. (2006). Long-term sequellae of oral appliance therapy in obstructive sleep apnea patients: Part 1. Cephalometric analysis. Am. J. Orthod. Dentofac. Orthop..

[27] Hammond R.J., Gotsopoulos H., Shen G., Petocz P., Cistulli P.A., Darendeliler M.A. (2007). A follow-up study of dental and skeletal changes associated with mandibular advancement splint use in obstructive sleep apnea. Am. J. Orthod. Dentofac. Orthop..

[28] Doff M.H., Hoekema A., Pruim G.J., Huddleston Slater J.J., Stegenga B. (2010). Long-term oral-appliance therapy in obstructive sleep apnea: A cephalometric study of craniofacial changes. J. Dent..

[29] Wang X., Gong X., Yu Z., Gao X., Zhao Y. (2015). Follow-up study of dental and skeletal changes in patients with obstructive sleep apnea and hypopnea syndrome with long-term treatment with the Silensor appliance. Am. J. Orthod. Dentofac. Orthop..

[30] Minagi H.O., Okuno K., Nohara K., Sakai T. (2018). Predictors of Side Effects with Long-Term Oral Appliance Therapy for Obstructive Sleep Apnea. J. Clin. Sleep Med..

[31] Hamoda M.M., Almeida F.R., Pliska B.T. (2019). Long-term side effects of sleep apnea treatment with oral appliances: Nature, magnitude and predictors of long-term changes. Sleep Med..

[32] Fransson A.M.C., Benavente-Lundahl C., Isacsson G. (2020). A prospective 10-year cephalometric follow-up study of patients with obstructive sleep apnea and snoring who used a mandibular protruding device. Am. J. Orthod. Dentofac. Orthop..

[33] Sakamoto Y., Furuhashi A., Komori E., Ishiyama H., Hasebe D., Sato K., Yuasa H. (2019). The Most Effective Amount of forwarding Movement for Oral Appliances for Obstructive Sleep Apnea: A Systematic Review. Int. J. Environ. Res. Public Health.

[34] Thurzo A., Urbanová W., Novák B., Waczulíková I., Varga I. (2022). Utilization of a 3D Printed Orthodontic Distalizer for Tooth-Borne Hybrid Treatment in Class II Unilateral Malocclusions. Materials.

